# Antigenotoxic Effects and Possible Mechanism of Red Yeast (*Sporidiobolus pararoseus*) on Aflatoxin B_1_-Induced Mutagenesis

**DOI:** 10.3390/biom11050734

**Published:** 2021-05-14

**Authors:** Romteera Kittichaiworakul, Sirinya Taya, Arpamas Chariyakornkul, Thanongsak Chaiyaso, Rawiwan Wongpoomchai

**Affiliations:** 1 Department of Biochemistry, Faculty of Medicine, Chiang Mai University, Chiang Mai 50200, Thailand; ployromteera.kiti@gmail.com (R.K.); arpamas.c@gmail.com (A.C.); 2 Functional Food Research Unit, Science and Technology Research Institute, Chiang Mai University, Chiang Mai 50200, Thailand; sirinya.t@cmu.ac.th; 3 Division of Biotechnology, Faculty of Agro-Industry, Chiang Mai University, Chiang Mai 50100, Thailand; thanongsak.c@cmu.ac.th

**Keywords:** Aflatoxin B1, cancer chemoprevention, rat liver micronucleus test, *Salmonella* mutation assay, *Sporidiobolus pararoseus*, xenobiotic metabolizing enzymes

## Abstract

Red yeast (*Sporidiobolus pararoseus*), obtained from glycerol waste in the biodiesel process, has been used as a mycotoxin sorbent in some agricultural products. This study focused on the antigenotoxic effects of red yeast on aflatoxin B_1_ (AFB_1_)-induced mutagenesis, using a *Salmonella* mutation assay and a rat liver micronucleus test. Red yeast was sequentially extracted to obtain hexane, acetone, hot water, and residue fractions. Carbohydrates were mainly found in hot water extract (HWE), while proteins were observed in the residue fraction. The amount of lycopene in hexane extract (HE) was higher than the amount of *β*-carotene in HE. All red yeast extracts were not mutagenic in the *Salmonella typhimurium* strains TA98 and TA100 under the presence and absence of metabolic activation. Among the extracts obtained from red yeast, HE presented the strongest antimutagenicity against AFB_1_-induced mutagenesis in both strains, but HWE did not show any antimutagenicity. The oral administration of red yeast, HE, and HWE for 28 days was further investigated in rats. These extracts did not induce micronucleated hepatocytes. Furthermore, they modulated the activities of some detoxifying enzymes but did not alter the activities of various cytochrome P450 isozymes. Notably, they significantly decreased hepatic micronucleus formation in AFB_1_-initiated rats. HE altered the activity of hepatic glutathione-*S*-transferase but did not affect its protein expression. Taken together, the antigenotoxicity of red yeast against AFB_1_-induced mutagenesis might be partly due to the modulation of some detoxifying enzymes in AFB_1_ metabolism. *β*-Carotene and lycopene might be promising antigenotoxic compounds in red yeast.

## 1. Introduction

The aflatoxins produced by Aspergillus species are the major contaminants in agricultural products such as peanuts, corn, and spices [1]. Aflatoxin B_1_ (AFB_1_) is the most potent carcinogen among aflatoxins that can cause hepatocellular carcinoma [2]. Several decontamination methods, including chemical treatment using alkalization and ammonization, and physical treatment using heating and irradiation, have been used to either reduce or eliminate AFB_1_ contamination in agricultural food. Nevertheless, they are impractical due to their cost, hazard, and limited efficacy [3]. The utilization of sorbent additives to prevent AFB_1_ absorption in the gastrointestinal tract has been used as one approach to resolve AFB_1_ contamination. It was recently found that yeast cell wall-based products were more effective than inorganic sorbents, due to their broad range of mycotoxin binding, biodegradation, and nutritional value. Moreover, the yeast cell wall increased the AFB_1_ elimination in the feces of ewes fed with an aflatoxin contaminated diet [4] and absorbed several mycotoxins in in vivo models [5,6].

Red yeast *(Sporidiobolus pararoseus)*, a single-cell microorganism belonging to the class Basidiomycota, was cultivated in crude glycerol waste from the biodiesel process [7]. Red yeast consists of numerous active compounds, including *β*-glucan, *β*-carotene, and several carotenoid derivatives [8]. Among the carotenoids, torulene and torulahodin exerted antioxidant, anti-inflammatory, anti-apoptotic, and anti-cancer activities [7,9,10]. *β*-Glucan, a major component in the red yeast cell wall, had multiple functions, such as immunomodulating [11] and antibacterial activities [12,13], and reduction of mycotoxin absorption [14]. Furthermore, cell wall manno-oligosaccharide also exhibited antimutagenicity and antioxidant activities [15].

Cancer is one of the most important health issues worldwide. The process of carcinogenesis can be divided into at least three stages, including initiation, promotion, and progression. The initiation stage is the first step that involves the alteration of genetic materials, resulting in the dysregulation of cell proliferation and cell death in subsequent processes [16]. Genotoxic effects in this stage refer to the result of a compound that injures genetic materials, including either DNA or the cellular components that control the integrity of the genome [17]. Therefore, all mutagens are genotoxic but all genotoxic agents are not mutagenic. However, some types of cancer can be prevented, due to their main causes being diet and lifestyle. Hence, cancer chemopreventive agents might primarily intervene in the process of carcinogenesis, particularly the initiation step to eliminate premalignant cells before they become malignant. Sources of cancer chemopreventive agents are not only found in plants and algae, but also in yeasts. Therefore, the present study aimed to investigate the genotoxicity and antigenotoxicity of red yeast and its ingredients using a *Salmonella* mutation assay and a rat liver micronucleus test. The inhibitory mechanisms of effective fractions of red yeast involving xenobiotic metabolizing enzymes were examined.

## 2. Materials and Methods

### 2.1. Chemicals

2-Aminoanthracene (2-AA) and 2-(2-furyl)-3-(5-nitro-2-furyl)-acrylamide (AF-2) were obtained from Wako pure chemicals (Osaka, Japan). Aflatoxin B_1_ (AFB_1_), *β*-carotene, lycopene, resorufin, ethoxyresorufin and methoxyresorufin, erythromycin, cytochrome C, reduced glutathione, and 2, 6-dichlorophenolindolephenol (DCPIP) were provided by Sigma-Aldrich (St. Louis, MO, USA). Uridine-5′-diphosphoglucuronic acid was purchased from US Biological (Salem, MA, USA). Collagenase type IV and 4′-6-diamidino-2-phenylindole (DAPI) were acquired from Gibco/Invitrogen Corp. (Waltham, MA, USA), and anti-GSTA1 antibody and anti-UGT1A1 were purchased from Abcam (Cambridge, UK), respectively. All other chemicals were at least analytical grade.

### 2.2. Preparation of Red Yeast Extracts

Red yeast (*S. pararoseus* KM281507) was cultivated in a medium consisting of 0.01% yeast extract, 5.50% crude glycerol, 0.55% KH_2_PO_4_, 0.53% (NH_4_)_2_SO_4_, 0.37% K_2_HPO_4_, 0.05% MgSO_4_ ∙7H_2_O, 0.02% MnSO_4_∙H_2_O, and 0.05% NaCl and fermented in an airlift bioreactor at 24 °C for 7 days [18]. The dried red yeast obtained from a vacuum spray dryer (5.09 ± 0.12% moisture) was suspended in hexane and lysed by glass bead pulverization with vortexing for 10 min. The resulting supernatant from centrifugation was evaporated and freeze-dried, obtaining the hexane extract. Subsequently, the resulting pellet was re-extracted with acetone under the same procedure to obtain the acetone extract. The remaining lower part was added to distilled water and heated at 121 °C for 20 min before the mixture was further centrifuged at 10,000 rpm for 10 min. Then, the upper part was collected and freeze-dried, obtaining the hot water extract. The lower part residue was dehydrated using a hot air oven at 55 °C.

### 2.3. Analysis of Chemical Constituent in Red Yeast

The content of total phenolic compounds was measured using a Folin–Ciocalteu assay described by Sankam et al. [19]. The sample and Folin–Ciocalteu reagent were mixed and incubated at 45 °C for 15 min. The absorbance at 750 nm was measured using a UV-visible spectrometer. The total phenolic content was calculated using a gallic acid standard curve and expressed as mg of gallic acid equivalents (GAE) per g of extract.

The content of total carbohydrates was determined with a phenol–sulfuric acid assay [20] using glucose as a standard. Various red yeast extracts were incubated with sulfuric acid at 90 °C for 30 min, followed by adding phenol solution, and the mixture was further incubated at room temperature for 5 min. The carbohydrate content was measured at 490 nm using a UV-visible spectrometer and calculated as mg of glucose per g of extract using the calibration curve of glucose.

The content of carotenoid derivatives was analyzed using reverse-phase HPLC according to the method of Shi et al. [8]. HPLC was carried out on a reverse phase C18 column (Agilent 4.6 mm × 250 mm, 5 µM). The mobile phase system consisted of a gradient composed of acetonitrile/water/formic acid (86:10:4 *v/v/v*) as phase A and ethyl acetate: formic acid (96:4 *v/v*) as phase B with a flow rate of 1 mL/min. The optical density at wavelengths of 338, 426, 452, and 478 nm was detected. The content of carotenoid derivatives was characterized and calculated using standard *β*-carotene and lycopene.

### 2.4. Mutagenicity and Antimutagenicity of Red Yeast Using Salmonella Mutation Assay

The mutagenicity of red yeast powder and its extracts, at concentrations ranging from 40 to 5000 µg/plate, was assessed using a *Salmonella* mutation assay according to the method of Inboot et al. [21]. *Salmonella typhimurium* tester strains TA98 and TA100 were kindly supplied by Dr. Kei-Ichi Sugiyama, National Institute of Health, Tokyo, Japan. AF-2 and 2-AA were used as standard mutagens in the absence (−S9) and presence (+S9) of metabolic activation, respectively. S9 fraction was prepared from 8–10 week-old male Wistar rat (*Rattus norvegicus*) injected with phenobarbital and β-naphthoflavone. Mutagenicity was expressed using the mutagenic index (MI) calculated from the number of revertant colonies divided by the number of spontaneous revertant colonies. The mutagenicity was classified when the MI value was over 2-fold.

The antimutagenicity test of red yeast powder and its extracts was modified from the previous procedure on the mutagenicity test. The concentrations of test compounds, ranging from 40 to 1000 ug/plate, were neither cytotoxic nor mutagenic to bacterial tester strains. AFB_1_ concentrations at 25.0 and 12.5 ng/plate were used as a positive mutagen in TA98 and TA100, respectively, under metabolic activation conditions. *β*-Carotene and lycopene, the possible constituents in red yeast, were also assessed for their antimutagenic activities against AFB_1_-induced mutagenesis. The percentage of inhibition of each sample was calculated as described by Inboot et al. [21].

### 2.5. Animals

Three-week old male Wistar rats (50–70 g body weight (bw)) were purchased from Nomura Siam International (Bangkok, Thailand). Rats were acclimatized for 1 week before starting the experiment. They were housed in controlled environments with a dark–light cycle of 12:12 h and at a temperature of 25 ± 1 °C. Water and basal diet were provided ad libitum. The protocol was approved by the Animal Ethic Committee of the Faculty of Medicine, Chiang Mai University (Approval number: 38/2560).

### 2.6. Clastogenicity and Anticlastogenicity of Red Yeast Using a Rat Liver Micronucleus Test

**Figure 1:**
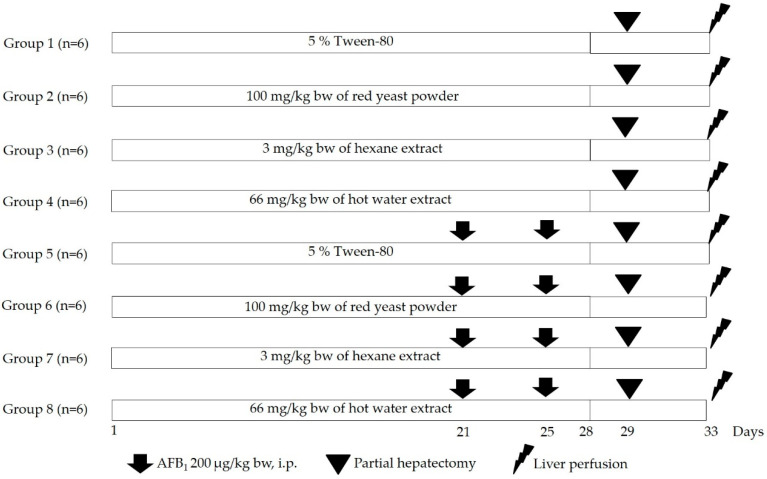
Experimental design for the study on clastogenicity and anticlastogenicity of red yeast and its extracts in rats.

Rats were randomly divided into 8 groups, 6 rats per group, as shown in Figure 1. Groups 1 to 4 received a solvent vehicle and were set up to evaluate the clastogenicity, while groups 5 to 8 were intraperitoneally injected with 200 µg/kg bw of AFB_1_ on days 21 and 25 of an experiment and were used for anticlastogenic assessment. Groups 1 and 5 were orally administrated with 5% Tween-80 as the negative and positive control groups, respectively. The other groups were fed with various doses of red yeast powder and its extracts for 28 consecutive days. On day 29, all rats were partially hepatectomized to amplify initiated hepatocytes. A liver section was collected from each rat to investigate xenobiotic metabolizing enzyme activity. On day 33, rats were anesthetized with thiopental and subjected to isolation of single hepatocytes using the collagenase perfusion method [22]. The number of micronuclei (MN), micronucleated hepatocytes (MNH), binucleated hepatocytes (BNH), and mitotic cells was counted under a fluorescent microscope, using 4′,6-diamidino-2-phenylindole (DAPI) staining. Approximately 2000 hepatocytes in at least 15 random fields with 400× magnification per rat were counted.

### 2.7. Determination of the Activities of Hepatic Phases I and II Xenobiotic Metabolizing Enzymes

The liver section was homogenized in a homogenizing buffer (pH 7.4) containing 1.15% *w/v* KCl and 0.25 mM phenylmethylsulfonyl fluoride (PMSF) and centrifuged at 10,000 rpm for 20 min at 4 °C. Then, the supernatant was further centrifuged at 30,000 rpm for 60 min to obtain the resulting supernatant as a cytosolic fraction and a pellet as a microsomal fraction. Total protein contents of both fractions were determined using the Lowry method.

The activities of cytochrome P450s (CYP) including CYP 1A1, CYP 1A2, and CYP 3A2 were obtained using the MROD, EROD, and ENDM methods, respectively, and according to Suwannakul et al. [23]. Ethoxyresorufin and methoxyresorufin were used as substrates in the CYP1A1 and CYP1A2 reactions, respectively. The excitation and emission wavelengths at 520 and 590 nm were measured and expressed as *f*mol/min/mg protein. Erythromycin was used as a substrate in the CYP3A2 enzymatic reaction, and Nash reagent was added in the last step. The CYP3A2 activity was determined at 550 nm using a spectrophotometer and expressed as *p*mol/min/mg protein.

NADPH-cytochrome P450 reductase (CPR) activity was estimated according to the method of Punvittayagul et al. [24]. The reaction mixture consisting of 0.3 M potassium phosphate buffer (pH 7.5), potassium cyanide, NADPH, and cytochrome C was incubated with a microsomal fraction at 37 °C for 2 min. The enzyme activity was measured by the reduction of cytochrome C at an optical density of 550 nm and using a molar extinction coefficient of 21 mM^−1^ cm^−1^ and was expressed as unit/mg protein.

Glutathione-*S*-transferase (GST) activity was determined using the slightly modified method of Sankam et al. [19]. Briefly, the reaction mixture containing 10 mM GSH, 0.2 M potassium phosphate pH 6.5, and CDNB was incubated with a cytosolic fraction at 37 °C for 20 min. The activity of GST expressed as unit per mg protein was measured at a wavelength of 340 nm using a spectrophotometer and calculated using a molar extinction coefficient of 9.6 mM^−1^ cm^−1^.

UDP-glucuronosyltransferase (UGT) activity was determined according to the method of Suwannakul et al. [23]. A microsomal fraction was added into a reaction mixture consisting of Tris-HCl, MgCl_2_, *p*-nitrophenol, and UGT and then incubated at 37 °C for 20 min. The enzymatic reaction was stopped with 10% TCA, and the upper part of the mixture was collected. The activity of UGT was measured at 405 nm using a spectrophotometer and expressed as unit/mg protein using a molar extinction coefficient of 21.0 mM^−1^ cm^−1^.

NADPH quinone oxidoreductase-1 (NQO-1) activity was determined as described elsewhere [24]. In brief, the reaction mixture containing Tris-HCl (pH 7.4), BSA, tween 20, NADPH and FAD, and a cytosolic fraction was mixed with DCPIP, and the absorbance at 600 nm was measured. The activity of NQO-1 was expressed as nmol/min/mg protein using a molar extinction coefficient of 21.0 mM^−1^ cm^−1^.

Heme oxygenase-1 (HO-1) activity was estimated using a slightly modified method of Punvittayagul et al. [24]. The reaction mixture containing NADPH, glucose-6-phosphate, glucose-6-phosphate dehydrogenase, hemin, and rat liver cytosol as a source of biliverdin reductase, was mixed with a microsomal fraction and incubated at 37 °C for 1 h. Then, the enzymatic reaction was stopped using chloroform. The activity of HO-1 was measured at wavelengths of 460 and 530 nm and expressed as nmol/min/mg protein using a molar extinction coefficient of 40 mM^−1^ cm^−1^.

### 2.8. Determination of Protein Expression of Xenobiotic Metabolizing Enzymes

The protein expression of xenobiotic metabolizing enzymes in liver was determined using Western blot analysis. Cytosolic proteins were separated by sodium dodecyl sulfate–polyacrylamide gel electrophoresis (SDS-PAGE) and transferred to nitrocellulose membrane, according to the method of Suwannakul et al. [23]. The membrane was incubated with Anti-GSTA1 antibody (1:5000 dilution) for 1 h. The membrane was further incubated with a secondary antibody consisting of peroxidase-conjugated anti-rabbit IgG (1:10,000 dilution) for 30 min. After microsomal proteins were separated, the specific proteins were blotted on the membrane. Then, the anti-UGT1A1 antibody (1:2500 dilution) was stained overnight and the peroxidase-conjugated anti-rabbit IgG (1:10,000 dilution) was used as the secondary antibody. The GSTA1 and UGT1A1 protein expressions were detected using horseradish peroxidase and exposed to film. The intensity value was normalized to β-actin and protein disulfide isomerase (PDI) for cytosolic and microsomal enzymes, respectively.

### 2.9. Statistical Analysis

The data from the *Salmonella* mutation assay were presented as mean ± SEM of three independent measurements. The data from animal experiments were expressed as mean ± SD of three independent experiments. The statistical significance of differences was analyzed by one-way ANOVA using SPSS version 23.0, and a *p*-value less than 0.05 was considered as a significant difference.

## 3. Results

### 3.1. Chemical Ingredients in Red Yeast

One kilogram of red yeast powder provided 30.0, 3.4, 662.7, and 166.4 g of hexane extract, acetone extract, hot water extract, and a residual portion, respectively. The chemical constituents of red yeast and its extracts are shown in Table 1. The major part of red yeast was carbohydrates, which were present in the hot water extract. The acetone extract and a residue fraction contained higher amounts of phenolic compounds than the other extracts, while hexane extract contained a high content of carotenoid derivatives, including *β*-carotene and lycopene. Furthermore, the residue from the extraction contained a high content of proteins.

### 3.2. Mutagenicity and Antimutagenicity of Red Yeast in Salmonella Mutation Assay

**Figure 2:**
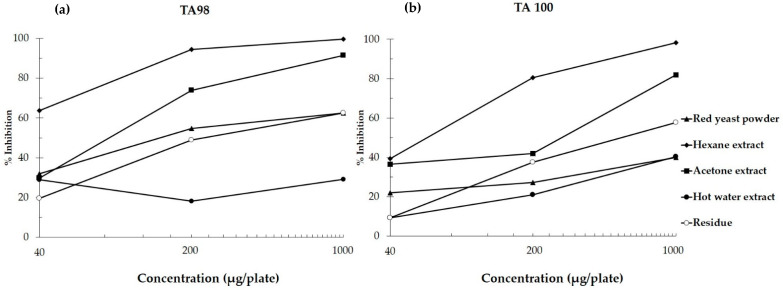
Antimutagenicity of red yeast and its extracts against AFB_1_-induced mutagenesis using *S. typhimurium* strains TA98 (**a**) and TA100 (**b**).

Red yeast and its extracts did not alter the number of revertant colonies of *S. typhimurium* TA98 and TA100 in the absence and presence of metabolic activation compared to their vehicle control. It was suggested that red yeast and its extracts were not mutagenic in the *Salmonella* mutation assay. However, hexane and acetone extracts at 5000 µg/plate suppressed the number of spontaneous revertant colonies indicating toxicity (Table 2). Non-cytotoxic doses of red yeast and its extracts were selected for a further antimutagenicity test. Red yeast could reduce the number of revertant colonies induced by AFB_1_ in both TA98 and TA100 strains. However, it showed milder antimutagenicity than its fractions including hexane, acetone, and residue parts. The hexane extract of red yeast exhibited a strong antimutagenic effect on AFB_1_-induced mutagenesis. Notably, the antimutagenicity of hot water extract of red yeast was not observed, with less than 30% inhibition in this bacterial mutation model (Figure 2).

Furthermore, some major compounds found in red yeast extracts were evaluated for their antimutagenicity. The estimated amounts of *β*-carotene and lycopene in hexane extract were assessed. *β*-Carotene at 20 ng/plate strongly decreased the mutagenicity of AFB_1_, with an approximate 80% inhibition in strains TA 98 and TA100, while lycopene at 100 ng/plate could moderately reduce the mutagenicity of AFB1, with a roughly 50% inhibition (Table 3). From these results, *β*-carotene was the most effective antimutagen against AFB_1_ mutagenicity in the bacterial mutation model.

### 3.3. Clastogenicity and Anticlastogenicity of Red Yeast in Rats

The clastogenicity and anticlastogenicity of red yeast powder and its attractive extracts were further evaluated using micronucleus formation in rats as the end point marker. The concentrations of hexane and hot water extracts used in this study were equivalent to their amounts in crude red yeast. Red yeast, hexane extract, and hot water extract did not induce the same number of micronucleated and binucleated cells and mitotic index in the liver tissue when compared with a vehicle control group. However, hexane extract significantly decreased the number of spontaneous binucleated hepatocytes (Table 4). Therefore, 100 mg/kg bw of red yeast and its active extracts were non-clastogenic in the liver of male rats.

An investigation of the anticlastogenicity of red yeast powder and its extracts against AFB_1_-induced micronucleus formation in rat liver was performed. AFB_1_ significantly increased the number of micronucleated hepatocytes, binucleated hepatocytes, and mitotic index when compared to a negative control. Interestingly, the oral administration of crude red yeast, hexane, and hot water extracts for 28 days significantly decreased the number of micronucleus and micronucleated hepatocytes in AFB_1_-initiated rats. Furthermore, red yeast and its hexane extract suppressed the number of binucleated hepatocytes in AFB_1_-treated rats. Thus, some compounds in hexane and hot water extracts of red yeast might be antimutagenic ingredients in red yeast.

### 3.4. Effect of Red Yeast on Xenobiotic Metabolizing Enzymes

**Figure 3:**
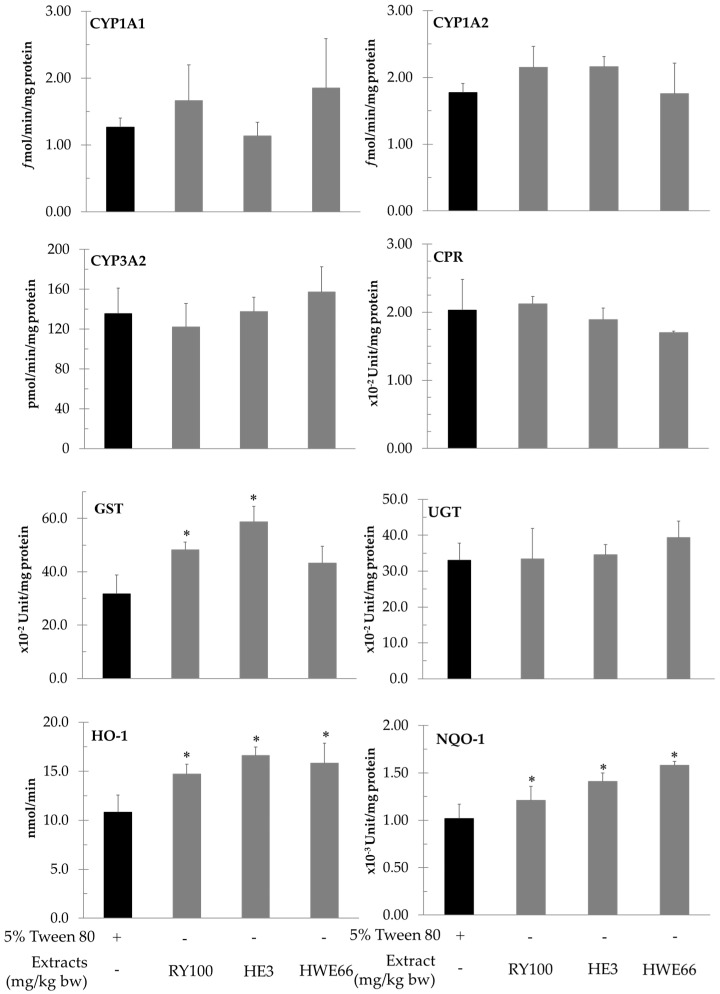
Effect of red yeast and its extracts on the activities of phase I and II xenobiotic metabolizing enzymes in rat liver. Values expressed as mean ± SD, n = 6. CYP: cytochrome P450; CPR cytochromeP450 reductase; GST: glutathione-*S*-transferase; HO-1: heme oxygenase; HE: hexane extract; HWE: hot water extract; NQO-1: NAD(P)H quinone oxidoreductase; RYP: red yeast powder; UGT: UDP-glucuronyltransferase. * Significantly different from 5%Tween80-treated rats (*p* < 0.05).

**Figure 4:**
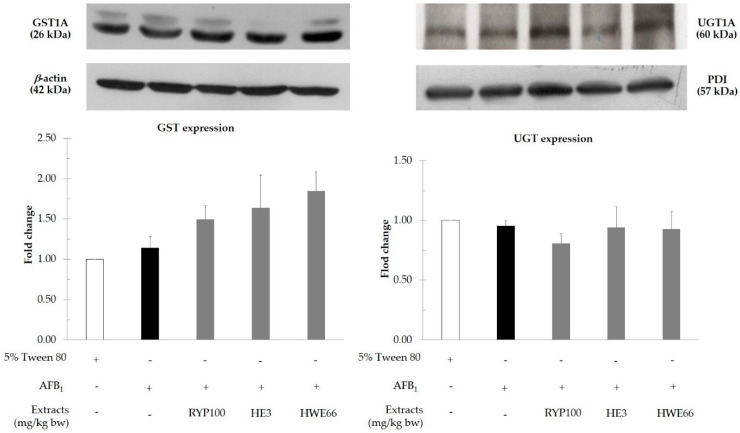
Protein expression of glutathione-*S*-transferase and UDP-glucuronosyltransferase in liver of AFB_1_-induced rats. HE: hexane extract; HWE: hot water extract; RYP: red yeast powder.

Red yeast and its polar and non-polar extracts modulated the activities of some phase II enzymes including GST, NQO-1, HO-1, and UGT but did not alter the activities of various cytochrome P450 isozymes in the liver of rats (Figure 3). The administration of both red yeast and hexane extract of red yeast significantly increased the activity of GST but did not alter its protein expression in the liver of AFB_1_-treated rats (Figure 4). However, hot water extract did not attenuate both phase I and II enzymes involved in AFB_1_ metabolism (Table 5).

## 4. Discussion

AFB_1_ contamination is an important food safety problem in post-harvest management. Chronic consumption of AFB_1_-contaminated food is a risk for liver cancer, particularly in Asia [25]. Red yeast is a product of yeast that has recently been introduced for use as an ingredient in animal feed. It belongs to the genus Sporidiobolus and could produce not only various prebiotic polysaccharides, which are commonly found in yeasts, but also carotenoids, which are different from common yeasts [4,15,26]. Our results found that red yeast possessed inhibitory effects on AFB_1_-induced genotoxicity in in vitro and in vivo models.

DNA damage is a pivotal cause of carcinogenesis. The avoidance of either food or environmental mutagens is still difficult to achieve. Therefore, consumption of cancer chemopreventive agents derived from natural products might be one reasonable approach for cancer prevention. This study used a range of genotoxicity testing methods such as a *Salmonella* mutation assay and an in vivo micronucleus assay to detect the antimutagenicity and anticlastogenicity of red yeast and its extracts. Red yeast and its extracts showed no mutagenic effects on *S. typhimurium* in strains TA98 and TA100, with and without metabolic activation. Several reports have shown that one group of phytonutrients in red yeast was carotenoids [27]. We found the hexane extract that contained at least two carotenoids, including *β*-carotene and lycopene, possessed the highest antimutagenic activity against AFB_1_-induced mutagenesis, when compared with crude red yeast and the other fractions. The antimutagenicity of *β*-carotene and lycopene proven in this study was in line with reports elsewhere [28]. *β*-Carotene and lycopene might inhibit the activation of some cytochrome P450 isoenzymes involved in AFB_1_ metabolism. Notably, hot water extract, which is a major part of red yeast, presented mild antimutagenicity in TA100 but did not affect TA98, with a lower than 30% inhibition. It was suggested that *β*-carotene and lycopene might be antimutagenic phytochemicals in red yeast, according to their antimutagenicity using a *Salmonella* mutation assay.

Genotoxicity is not only caused by DNA mutation but is also involved in chromosomal alteration. When chromosomal fragments occur, they are not able to be incorporated into the daughter nucleus during cell division, leading to micronucleus formation [29,30]. Our investigation found that red yeast and its extracts did not induce hepatic micronucleus formation but could diminish the number of micronuclei in the liver of AFB_1_-treated rats. Hexane extract exhibited the strongest anticlastogenicity in this animal model, which was correlated to its antimutagenic results in the bacterial mutation assay. However, the anticlastogenicity of hot water extract found in this study was not relevant to the antimutagenic outcome using the *Salmonella* mutation model. It is possible that the anticlastogenic ingredients in hot water extract were large molecules, such as oligosaccharides, that require digestive enzymes for absorption into enterocytes prior to acting on their target cells, including hepatocytes.

Generally, our body provides xenobiotic metabolizing enzymes to increase xenobiotic polarity, leading to either detoxification or intoxication of these foreign compounds. The current study found red yeast and its fractions did not alter the cytochrome P450 involving system but they could modulate the activities of some phase II xenobiotic metabolizing enzymes. The hexane extract of red yeast significantly increased the activity of GST, but not its protein expression, in AFB_1_-initiated rats. It was suggested that these hydrophobic molecules might allosterically regulate GST function in the liver. GST plays a vital role in the detoxifying fate of AFB_1_, due to its epoxide metabolites after biotransformation of the phase I metabolizing enzyme system [31]. Furthermore, red yeast, including its hydrophilic and hydrophobic parts, did not alter the activity of hepatic UGT in the liver of AFB_1_-treated rats. Although UGT has been known as the major phase II metabolizing enzyme in many drugs and toxicants, it does not act as an essential detoxifying enzyme of AFB_1_ [32]. These facts might be a reason why both red yeast and its hexane extract could forcefully reduce micronucleus formation in the liver of AFB_1_-treated rats.

Although the major part of red yeast is hydrophilic molecules, we found that the minute hydrophobic part of red yeast showed a more potent antigenotoxicity against AFB_1_-induced mutagenesis. Several studies have reported that *β*-carotene and lycopene showed an ability to reduce the mutagenic effect of AFB_1_ via activation and detoxification processes [33,34,35]. *β*-Carotene exhibited a protective effect on liver damage and against carcinogenesis induced by AFB_1_. Moreover, *β*-carotene could increase the activity of some detoxifying enzymes, such as GST, leading to reducing AFB_1_ toxicity in in vivo models [36]. Furthermore, lycopene modulated the activities of various detoxifying enzymes, such as GST, NQO-1, and HO-1 in rat liver [37,38,39]. It reduced AFB_1_ toxicity by enhancing GST and NQO expression thought Nrf-2 and ARE activations, respectively [38,40,41]. Our study suggested carotenoids, particularly *β-*carotene, might act as promising antigenotoxic compounds in red yeast.

## 5. Conclusions

In conclusion, red yeast exhibited an antigenotoxic potential on aflatoxin B_1_-induced mutagenesis using a *Salmonella* mutation assay and a rat liver micronucleus test. The inhibitory mechanism of red yeast might be involved in the modulation of xenobiotic metabolizing enzymes in aflatoxin B_1_ metabolism. *β*-Carotene and lycopene were considered as potential cancer chemopreventive agents in red yeast. This study suggests that red yeast might be an alternative source for cancer chemoprevention, particularly at the initiation stage of aflatoxin B_1_-induced carcinogenesis.

## Figures and Tables

**Table 1:** Chemical constituents of red yeast and its extracts.

Compounds	Contents (mg/g Extract)				
	Total Carbohydrate ^a^	Protein ^a^	Phenolic Compounds ^a^	Lycopene ^b^	*β*-Carotene ^b^
Red yeast powder	497.7 ± 12.6	62.3 ± 6.7	4.9 ± 0.4	0.024 ± 0.000	0.013 ± 0.001
Hexane extract	36.1 ± 0.0	12.0 ± 5.3	5.0 ± 1.1	0.449 ± 0.035	0.094 ± 0.009
Acetone extract	118.6 ± 14.3	8.8 ± 1.7	10.7 ± 0.0	0.0 ± 0.0	0.0 ± 0.0
Hot water extract	835.7 ± 64.6	22.9 ± 0.6	1.5 ± 0.0	0.0 ± 0.0	0.0 ± 0.0
Residue	192.0 ± 13.1	118.5 ± 2.1	11.2 ± 0.8	0.0 ± 0.0	0.0 ± 0.0

Values are presented as mean ± SD. ^a^ Detected by spectrophotometry ^b^ Detected by HPLC analysis.

**Table 2:** Mutagenicity of red yeast and its extracts in *S. typhimurium* strains TA98 and TA100 in the absence and presence of metabolic activation.

Treatment	Concentration (per Plate)	Number of His^+^ Revertant Colonies (Mutagenic Index)			
		TA98		TA100	
		+S9	−S9	+S9	−S9
DMSO	50 µL	29 ± 1 (1.00)	24 ± 2 (1.00)	136 ± 13 (1.00)	92 ± 3 (1.00)
DW	50 µL	28 ± 4 (1.00)	24 ± 1 (1.00)	136 ± 23 (1.00)	103 ± 5 (1.00)
2AA	0.05 µg	871 ± 157 (29.29)	-	815 ± 98 (6.07)	-
AF-2	0.10 µg	-	264 ± 11 (11.01)	-	-
AF-2	0.01 µg	-	-	-	917 ± 14 (10.00)
Red yeast powder	40 µg	26 ± 3 (0.80)	22 ± 2 (0.90)	125 ± 22 (0.90)	84 ± 8 (0.93)
	200 µg	23 ± 2 (0.77)	22 ± 1 (0.89)	103 ± 13 (0.75)	85 ± 11 (0.95)
	1000 µg	28 ± 4 (0.95)	19 ± 1 (0.77)	113 ± 7 (0.84)	99 ± 6 (1.09)
	5000 µg	27 ± 5 (0.90)	22 ± 0 (0.91)	79 ± 13 (0.81)	103 ± 6 (1.15)
Hexane extract	40 µg	27 ± 3 (0.90)	22 ± 3 (0.89)	125 ± 19 (0.91)	74 ± 5 (1.81)
	200 µg	25 ± 3 (0.84)	24 ± 2 (0.97)	106 ± 9 (0.78)	83 ± 4 (0.91)
	1000 µg	23 ± 1 (0.77)	23 ± 2 (0.94)	88 ± 7 (0.66)	77 ± 6 (0.85)
	5000 µg	19 ± 2 (0.66) ^K^	14 ± 0 (0.59) ^K^	78 ± 8 (0.57) ^K^	66 ± 1 (0.73) ^K^
Acetone extract	40 µg	28 ± 4 (0.95)	25 ± 3 (1.02)	128 ± 20 (0.93)	84 ± 2 (0.84)
	200 µg	28 ± 5 (0.94)	25 ± 4 (1.03)	131 ± 9 (0.96)	90 ± 3 (0.99)
	1000 µg	29 ± 3 (0.98)	23 ± 2 (0.95)	95 ± 12 (0.70)	88 ± 10 (0.98)
	5000 µg	19 ± 2 (0.64) ^K^	20 ± 1 (0.81)	92 ± 7 (0.68) ^K^	86 ± 2 (0.95)
Hot water extract	40 µg	29 ± 4 (1.06)	23 ± 1 (0.98)	137 ± 16 (1.03)	98 ± 6 (0.96)
	200 µg	26 ± 2 (0.93)	24 ± 2 (1.03)	150 ± 11 (1.15)	107 ± 13 (1.07)
	1000 µg	27 ± 3 (0.97)	23 ± 1 (0.98)	143 ± 23 (1.06)	93 ± 9 (0.92)
	5000 µg	26 ± 3 (0.95)	21 ± 1 (0.89)	161 ± 35 (1.18)	92 ± 13(0.91)
Residue	40 µg	28 ± 2 (0.95)	24 ± 1 (0.97)	122 ± 16 (0.89)	86 ± 2 (0.95)
	200 µg	21 ± 1 (0.73)	22 ± 1 (0.92)	121 ± 17 (0.89)	86 ± 4 (0.95)
	1000 µg	28 ± 3 (0.96)	23 ± 3 (0.95)	116 ± 6 (0.86)	88 ± 1 (0.97)
	5000 µg	30 ± 3 (1.03)	24 ± 2 (0.99)	110 ± 5 (0.80)	79 ± 2 (0.87)

Values are presented as mean ± SEM. 2AA: 2-Aminoanthracene, AF-2: 2-(2-furyl)-3-(5-nitro2-furyl)-acrylamide; DMSO: dimethyl sulfoxide; DW: distilled water; K: killing effect.

**Table 3:** Antimutagenicity of some compounds found in red yeast against AFB_1_-induced mutagenesis using *S. typhimurium* strains TA98 and TA100.

Treatment	Concentration (per Plate)	TA98		TA100	
		Number of Revertant Colonies	%Inhibition	Number of Revertant Colonies	%Inhibition
DMSO	50 µL	30 ± 7	-	118 ± 6	-
AFB_1_	1.25 ng	1148 ± 30 *	-	893 ± 42 *	-
*β*-carotene	20 ng	283 ± 27 **	77.4	293 ± 37 **	78.4
Lycopene	1000 ng	526 ± 25 **	55.6	540 ± 16 **	48.4

Values are expressed as mean ± SEM * Significantly different from DMSO-treated group (*p* < 0.05) ** Significantly different from AFB_1_-treated group (*p* < 0.05).

**Table 4:** Clastogenicity and anticlastogenicity of red yeast and its extracts using rat liver micronucleus assay.

Treatment	Final Body Weight (g)	Number per 1000 Hepatocytes			Mitotic Index	%Inhibition
		MNH	MN	BNH Cells		
Vehicle	276 ± 18	3.9 ± 0.5	3.9 ± 0.5	1.52 ± 0.2	0.64 ± 0.1	-
Vehicle + RYP 100 mg/kg bw	262 ± 13	3.5 ± 0.7	3.6 ± 0.8	1.52 ± 0.3	0.73 ± 0.1	-
Vehicle + HE 3 mg/kg bw	277 ± 23	3.3 ± 0.5	3.6 ± 0.6	0.89 ± 0.2 *	0.79 ± 0.1	-
Vehicle + HWE 66 mg/kg bw	276 ± 18	3.4 ± 0.8	3.5 ± 0.8	1.56 ± 0.6	0.57 ± 0.1	-
AFB_1_	270 ± 15	13.3 ± 2.1 *	13.6 ± 2.0 *	2.28 ± 0.3 *	1.29 ± 0.3 *	-
AFB_1_ + RYP 100 mg/kg bw	288 ± 8	7.1 ± 1.6 **	7.3 ± 1.3 **	1.71 ± 0.4 **	1.28 ± 0.2	46.4 ± 12.0
AFB_1_ + HE 3 mg/kg bw	291 ± 16	6.0 ± 2.1 **	6.2 ± 2.0 **	1.70 ± 0.2 **	1.27 ± 0.3	54.6 ± 15.7
AFB_1_ + HWE 66 mg/kg bw	288 ± 10	9.2 ± 1.7 **	9.3 ± 1.7 **	1.93 ± 0.4	1.48 ± 0.1	26.5 ± 7.7

Values are presented as mean ± SD. AFB_1_: aflatoxin B_1_; BNH: binucleated hepatocytes; HE: hexane extract; HWE: hot water extract; MN: micronucleus; MNH: micronucleated hepatocytes; RYP: red yeast powder; Vehicle:5% Tween80. * significantly different from 5%Tween80-treated rats (*p* < 0.05); ** significantly different from AFB_1_-treated rats (*p* < 0.05).

**Table 5:** Effect of red yeast and its extracts on the activities of phase I and II xenobiotic metabolizing enzymes in the liver of AFB_1_-induced rats

Enzyme Activities (per mg Protein)	5% Tween-80	AFB_1_	AFB_1_+ RYP 100 mg/kg bw	AFB_1_ + HE 3 mg/kg bw	AFB_1_ + HWE 66 mg/kg bw
Cytochrome P450 1A1 (*f*mol/min)	1.30 ± 0.51	1.05 ± 0.25	1.19 ± 0.48	0.95 ± 0.14	0.92 ± 0.20
Cytochrome P450 1A2 (*f*mol/min)	0.55 ± 0.02	0.47 ± 0.11	0.61 ± 0.19	0.56 ± 0.04	0.60 ± 0.15
Cytochrome P450 3A2 (*p*mol/min)	113.56 ± 14.98	134.30 ± 15.86	108.98 ± 16.72	141.35 ± 32.38	122.49 ± 10.16
Heme oxygenase (nmol/min)	9.74 ± 0.55	10.42 ± 2.83	10.08 ± 1.03	9.42 ± 1.82	9.84 ± 1.78
NADPH quinone reductase (×10^−3^ Unit)	1.37 ± 0.22	1.65 ± 0.12 *	1.71 ± 0.13	1.69 ± 0.13	1.46 ± 0.26
NADPH-Cytochrome P450 reductase (×10^−3^ Unit)	2.42 ± 0.13	2.58 ± 0.34	2.26 ± 0.41	2.29 ± 0.30	2.52 ± 0.35
Glutathione-*S*-transferase (×10^−2^ Unit)	34.17 ± 4.28	47.24 ± 2.93 *	52.04 ± 1.66 **	59.84 ± 3.19 **	47.23 ± 4.58
UDP-glucuronyltransferase (×10^−2^ Unit)	34.40 ± 2.80	32.50 ± 5.10	31.10 ± 3.0	33.10 ± 2.50	36.20 ± 3.20

Values are presented as mean ± SD. HE: hexane extract; HWE: hot water extract; RYP: red yeast powder. * significantly different from 5%Tween80-treated rats (*p* < 0.05). ** significantly different from AFB_1_-treated rats (*p* < 0.05).

## Data Availability

Not applicable.
